# The Signaling Network Controlling *C. elegans* Vulval Cell Fate Patterning

**DOI:** 10.3390/jdb6040030

**Published:** 2018-12-11

**Authors:** Hanna Shin, David J. Reiner

**Affiliations:** 1 Institute of Biosciences and Technology, Texas A&M Health Science Center, Houston, TX 77030, USA; hshin2@medicine.tamhsc.edu; 2 College of Medicine, Texas A & M University, Houston, TX 77030, USA

**Keywords:** EGF, EGFR, Ras, Notch, DSL, Raf, RalGEF, Ral, signal transduction, MAP kinase

## Abstract

EGF, emitted by the Anchor Cell, patterns six equipotent *C. elegans* vulval precursor cells to assume a precise array of three cell fates with high fidelity. A group of core and modulatory signaling cascades forms a signaling network that demonstrates plasticity during the transition from naïve to terminally differentiated cells. In this review, we summarize the history of classical developmental manipulations and molecular genetics experiments that led to our understanding of the signals governing this process, and discuss principles of signal transduction and developmental biology that have emerged from these studies.

## 1. C. *elegans* Vulval Development

The *C. elegans* vulva is a textbook system for the study of developmental biology and signal transduction. The completed vulva is an epithelial tube that connects the uterus to the outside of the hermaphrodite: the vulva mediates egg-laying and mating with males. Importantly from the perspective of a developmental geneticist, the vulva is dispensable for viability. In vulvaless hermaphrodites, self-fertilized eggs hatch within the mother to produce live progeny. Consequently, this system is amenable to genetic manipulation, with genetic perturbations resulting in visible phenotypes such as Multivulva (Muv) and Vulvaless (Vul) [1].

**Figure 1:**
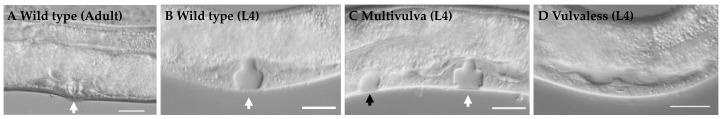
Images of wild type and mutant vulvae at adult or L4 stage. (**A**–**D**) White and black arrows indicate normal and ectopic pseudovulvae, respectively. (scale bar = 10 µm in (**A**) and 20 µm in (**B**–**D**)) (**A**) Vulva in wild type (N2) adult. (**B**) Vulva in wild type (N2) L4 stage. (**C**) Multivulva phenotype in *let-60* (*n1046*gf). (**D**) Vulvaless phenotype in *lin-12* (*n379*d).

During early larval development, the six vulval precursor cells (VPCs; also known as the Pn.p cells, P3.p–P8.p) are generated to form the vulval equivalence group. These cells are roughly equipotent, with any VPC capable of assuming any of the three potential VPC fates, 1°, 2°, or 3° (called primary, secondary, or tertiary). The VPCs are induced during the third larval (L3) stage. After initial patterning, the 22 daughter cells (eight cells from P6.p and seven cells from each P5.p and P7.p) form the vulva [2]. The final positioning of the vulva is ventral, at the anteroposterior and left-right mid-point (Figure 1).

In this review, we focus on the signaling network that governs developmental patterning of VPCs fates. Other important features of vulval development are outside the scope of this review, and are covered elsewhere [2]. For example, generation of the VPCs and establishment of competency occur before the events discussed are not described in this review. The timing of vulva development is controlled by the well-studied heterochronic system [3]. Generation of VPC lineages is relatively under-studied, beyond a sketch of a transcriptional gene regulatory network [4,5,6]. Polarity of 2° vulval lineages is controlled by overlapping Wnt systems [7,8,9]. Vulval morphogenesis is also relatively under-studied, though an interesting start has been made [10,11,12].

## 2. VPC Fate Patterning

Pattern formation of *C. elegans* vulval cell fates has proved to be an excellent model for the study of cell-cell communication. A confluence of research using the *C. elegans* VPCs, the *Drosophila* R7 photoreceptor, and mammalian cell culture and biochemistry led to the first consensus description of an intercellular signal, from ligand to nucleus. This signal is EGF (Epidermal Growth Factor)-EGFR (EGF Receptor) signaling through the Ras proto-oncogene activation of the Raf-MEK-ERK canonical MAP kinase cascade, which is the main 1°-promoting signal in VPC patterning. Also of great impact was the characterization of the Notch receptor signaling system, which is the main 2°-promoting signal in VPC fate patterning. Thus, VPC patterning holds a central place in the history of cell-cell signaling research in both development and cancer [13,14]. Here we discuss an updated view of the signaling network that patterns VPC fate.

**Figure 2:**
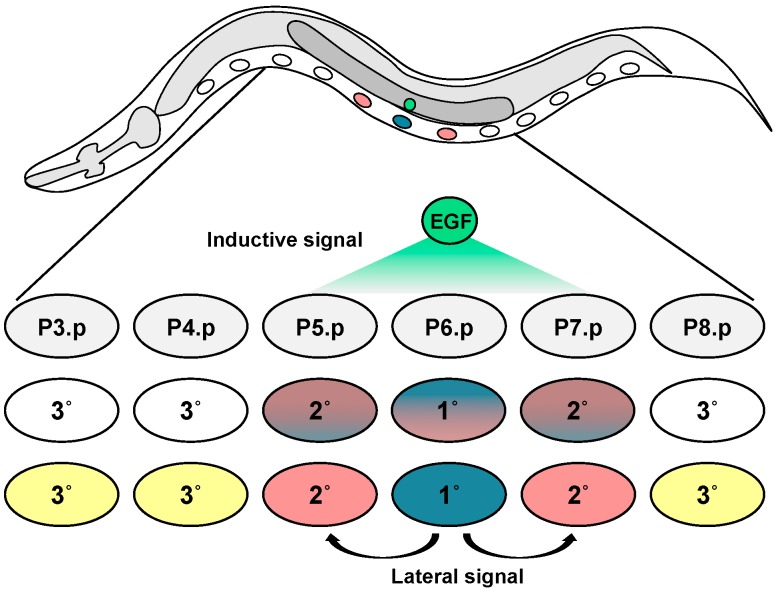
Overview of the *C. elegans* VPC fate patterning. The six naïve VPCs are numbered P3.p through P8.p. P6.p, closest to the Anchor Cell (AC), receives the highest level of EGF inductive signal and assumes 1° fate. P5.p and P7.p receives lower levels of inductive signal and lateral Notch signal from the P6.p to assume 2° fate. P3.p, P4.p, and P8.p receive insufficient inductive and lateral signals and adopt nonvulval fates.

The vulval equivalence group consists of six equipotent VPCs, arranged anteriorly to posteriorly along the ventral midline. These specialized cells are part of the epithelium (termed the “hypodermis” in *C. elegans*). During the L3 stage, the final pattern of 3°-3°-2°-1°-2°-3° cell fates is induced. The 1° and 2° cells are induced vulval fates: these VPCs go on to form the vulva after characteristic cell division lineages. The 3° cells are the “uninduced” or “ground” cell fate. 3° cells divide once and then fuse with the surrounding syncytial epithelium (Figure 2; [15]). This pattern occurs with 99.8% accuracy and the resulting cell lineages are invariant [16]. This pattern is induced by a signal from the Anchor Cell (AC), part of the somatic gonad, plus signals among the VPCs [17,18]. Ablation of the gonad during L1 stage, or the AC before the L3 stage, caused all VPCs to adopt 3° fate and fail to develop the vulva. The first detectable event of AC induction is positioning of the VPCs relative to the AC. P6.p, the presumptive 1° cell, becomes centered next to the AC [19]. Classical developmental biology experiments followed by decades of molecular genetics analysis has led to three non-exclusive mechanistic models that describe VPC fate patterning. Here we discuss the signaling network that generates the pattern of VPC fate.

### 2.1. The Morphogen Gradient Model Becomes the Graded Signal Plus Lateral Signal Model.

**Figure 3:**
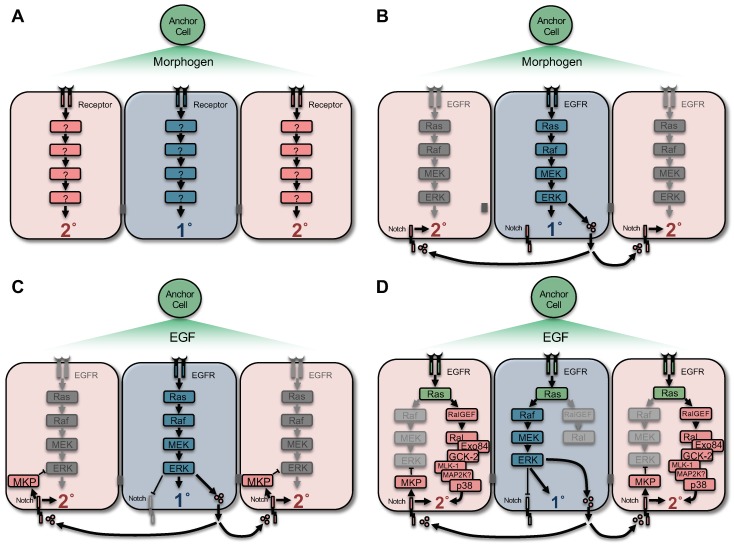
Models of *C. elegans* VPC fate patterning. (**A**) The Morphogen Gradient Model. Equipotent VPCs are patterned, by graded LIN-3/EGF (Morphogen) from the anchor cell ((**A**), (**C**)) through the activation of LET-23/EGFR (Receptor) based on position within the gradient. Yet in the absence of LIN-12/Notch the gradient cannot induce 2° fate, leading to the a Graded Signal plus Lateral Signal Model. (**B**) The Sequential Induction Model. In the response to the LIN-3, LET-23 activates the LET-60/Ras-LIN-45/Raf-MEK-2/MEK-MPK-1/ERK canonical MAP kinase signaling cascade to promote 1° fate. The induced presumptive 1° cells synthesizes redundant DSL/Notch ligands to laterally signal LIN-12/Notch activation to induce neighboring cells to assume 2° fate. (**C**) Mutual Antagonism. Additional antagonistic mechanisms prevent VPCs from adopting inappropriate cell fates. In the presumptive 1° cell, activation of LET-23/EGFR causes internalization and degradation of LIN-12/Notch. In the presumptive 2° cells, the LIN-12 transcriptional target *lip-1* is expressed. *lip-1* encodes ERK phosphatase (a DUSP MAP kinase phosphatase; MKP) to abrogate MPK-1/ERK activation in presumptive 2° cells. (**D**) The Graded Signal, Lateral Signal and and Sequential Induction Models were reconciled (Zand et al., 2011): all are thought to occur to pattern the VPC fates. During vulval fate patterning, LET-60/Ras switches effectors, from canonical LIN-45/Raf, which is necessary and sufficient for 1° fate induction, to non-canonical RGL-1/RalGEF-RAL-1/Ral that promotes 2° fate via activation of a GCK-2/MAP4K-PMK-1/p38 MAP kinase cascade in support of LIN-12/Notch [71]. The mechanism of this switch is unknown.

Combining cell lineage analysis with ablation of selected cells with a laser microbeam revealed the presence of cell-cell signaling events between the AC and VPCs and among VPCs [1,17,18,20,21]. From an elegant combination of these approaches arose the Morphogen Gradient Model (Figure 3A). The AC induces equipotent VPCs to assume their fate. P6.p, the VPC closest to the AC, typically becomes 1° [15,18]. Isolated VPCs (generated by ablation of other VPCs with a laser microbeam) assume 1° or 2° fate based on distance from the source of signal; VPCs close to the AC become 1°, while those distal from the AC become 2° [20]. This observation led to the model that it is dose of a “morphogen” signal that dictates VPC fate.

Evidence from mutants challenged the Morphogen Gradient Model. An extensive collection of mutations was generated that perturbed patterning in distinctive ways [22,23,24]. Loss of LIN-12/Notch function precluded induction of 2° fates in double mutant and cell manipulation experiments [24,25], thus arguing against a solely gradient-based model [26]. *lin-12* encodes a *C. elegans* Notch receptor [27], which, in combination with genetic data, indicates a lateral signaling role for LIN-12/Notch, as is found in many other systems. SUR-2/MED23 is required for 2° fate induction [28], and was later shown to be required for expression of the DSL ligands of Notch in the presumptive 1° cells [29]. Similarly, mutation of the *lin-4* heterochronic gene, required for activation of LIN-12/Notch, also blocks 2° fate induction [30]. Consequently, the Morphogen Gradient Model was replaced by a combination of Graded Signal plus Lateral Signal.

Further evidence of a graded signal came from experiments controlling dose of EGF and EGFR. *lin-3* and *let-23* are essential for 1° fate, and encode proteins similar to EGF and EGFR, respectively [31,32]. LIN-3/EGF is expressed in the AC during the induction of VPCs at L2 to L4 stages and is required in the AC for VPC induction [32,33,34]. Ectopic expression of LIN-3 is sufficient to induce VPCs in the absence of gonad, indicating that LIN-3 is also sufficient to induce 1° fate [32]. Again in isolated VPCs, 1° or 2° fate was induced by LIN-3 and LET-23/EGFR signaling dose, manipulated by genetic or transgenic means [35,36]. The presence of a gradient was later validated visually using a transgenic molecular marker: the P*_egl-17_::gfp* transcriptional fusion is a 1° fate marker that expresses GFP in induced 1° cells [37,38]. A more sensitive P*_egl-17_::cfp::lacZ* transcriptional reporter revealed a transient CFP signal in presumptive 2° cells. This weaker signal is sustained in the 2° lineages when negative regulators of 1° signaling are perturbed [39,40].

Taken together, these results indicate that a spatially graded signal is detected by VPCs: this graded signal contributes to the 3°-3°-2°-1°-2°-3° VPC fate pattern, the morphogen is the EGF ortholog LIN-3, and its receptor is the EGFR ortholog, LET-23, and LIN-12/Notch is required to signally laterally from the presumptive 1° cell, to induce 2° fate [41]. 

### 2.2. The Sequential Induction Model

In the Sequential Induction Model, molecular genetic characterization of core signaling components suggested that induction of 1° fate occurs first, then 2° fate ([42]; Figure 3B). Extensive mutant screens identified genes that are necessary and sufficient for 1° and 2° fate [22,24]. Molecular cloning and analysis of these genes, plus epistatic ordering of genes into pathways, identified a necessary and sufficient cascade for inducing primary 1° fate. Via the SEM-5/Grb adaptor and SOS-1/Sos Ras exchange factor, LET-23/EGFR activates the LET-60/Ras-LIN-45/Raf-MEK-2/MEK-MPK-1/ERK canonical MAP kinase cascade to induce 1° fate [43,44,45,46,47,48,49,50]. Screens for suppression of the Muv phenotype caused by activated LET-60/Ras discovered proteins now accepted as components of Ras-Raf-MEK-ERK signaling: SOC-2/SUR-8 is thought to function as a scaffold for LET-60/Ras-LIN-45/Raf [51,52] and KSR-1 is thought to function as a scaffold for LIN-45/Raf-MEK-2/MEK-MPK-1/ERK [53,54,55]. Thus, this highly conserved cascade in necessary and sufficient to induce 1° fate.

Critical for the induction of 2° fate is the Notch ortholog, LIN-12 [24,27], which mediates the lateral signal from presumptive 1° cell, P6.p, to presumptive 2° cells, P5.p and P7.p [25,56]. Three DSL Notch ligands, LAG-2, APX-1 and DSL-1, are synthesized in the presumptive 1° cell in response to inductive signal. These ligands are redundantly required to laterally signal the neighboring P5.p or P7.p to become 2° cells [29,57].

Genetic mosaic experiments showed expression of *let-23(*+*)* in P6.p, but not in P5.p and P7.p, supported normal vulva induction [42,58]. Coupled with the synthesis of DSL ligands for LIN-12 in presumptive 1° cells, these results are consistent with the Sequential Induction. This stepwise signaling—first 1°, then 2°—was considered to be inconsistent with the Graded Signal Model, and was the subject of vigorous debate at conferences.

### 2.3. Mutual Antagonism

A key mechanism by which the VPCs are accurately patterned is what we term “Mutual Antagonism”. Though they start as equipotent, initially specified VPCs alter their signaling network to exclude signals that promote the opposing fate (also see “Transcriptional reprogramming of the VPC signaling network”, below). This feature of the signaling network likely reduces conflicting signals, and thus the rate of the VPCs committing to inappropriate or ambiguous cell fates. In turn, by decreasing formation of aberrantly patterned vulvae, this network feature likely increases the reproductive fitness of the animal.

Multiple lines of evidence point to an antagonistic tension between presumptive 1° and 2° cells (illustrated in Figure 3C). Prior to induction in L2 and early L3 stages, all six VPCs express LIN-12 [59]. Upon induction, in the developing 1° cell LIN-12 is internalized and degraded [59,60,61]. The mechanism of LIN-12 down-regulation is as yet unknown, but it depends on the MPK-1/ERK 1°-promoting target, SUR-2 of the Mediator complex [62].

In addition, the 1°-promoting LET-23-LET-60-LIN-45-MEK-2-MPK-1 canonical MAP kinase cascade is inhibited in specified 2° cells, P5.p and P7.p. After induction, transcription of the ERK phosphatase, the dual-specificity phosphatase (DUSP) MAP kinase phosphatase (MKP) LIP-1, is induced by LIN-12/Notch signaling in these cells [37]. The *egl-17* gene is a transcriptional target of 1°-promoting MPK-1, which in turn inhibits the LIN-1/Ets transcription factor [38,63]. In wild-type VPCs, a transient pulse of *egl-17* transcriptional reporter can be observed in presumptive 2° cells [39]. In the absence of LIP-1 and other LIN-12 transcriptional client genes, the *lst* genes, *dpy-23* and *ark-1* (see below), the signal from the *egl-17* reporter persists [37,39]. In addition to LIP-1, the DEP-1 receptor tyrosine phosphatase, predicted to inhibit LET-23 activity, is expressed in 2° cells after induction to antagonize the 1°-promoting signal [64]. Conversely, the AGE-1/PI3K-PDK-1 signal functions to positively modulate 1° signal in VPCs [65].

Thus, in response to signaling cascades necessary for 1° and 2° fate, each cell type enacts programs to exclude promotion of the competing cell fate. A series of orphan 1°- and 2°- antagonizing “modifier genes” have been identified, but not placed functionally in the VPC patterning network [66,67]. These gene products could provide yet additional layers of Mutual Antagonism mechanisms. Perturbation of multiple antagonistic mechanisms confers patterning errors, suggesting that, collectively, these Mutual Antagonism mechanisms are critical for accurate VPC fate patterning.

### 2.4. Reconciling the Sequential Induction and Graded Signal Models

The “Sequential Induction” model does not explain how graded EGF signal promotes 2° fate or varying levels of LIN-3 and LET-23 signaling dose result in different signaling outcomes. This contradiction remained in the field for 16 years [41]. However, work from our lab reconciled these two models by showing that graded LIN-3-LET-23 signaling promotes 2° fate through LET-60/Ras switching downstream effectors. In contrast to the LET-60-LIN-45-MEK-2-MPK-1 cascade that is necessary and sufficient to induce 1° fate, the LET-60-RGL-1/RalGEF-RAL-1/Ral signal promotes 2° fate as a positive modulator in support of LIN-12 ([68,69]; Figure 3D). RalGEF-Ral is a proto-oncogenic non-canonical Ras effector in human cells (reviewed in [70]). We further investigated downstream of RAL-1 and found that RAL-1 signals through EXOC-8/Exo84-GCK-2/MAP4K-PMK-1/p38 MAPK to promote 2° fate [71]. Yet while LET-60 effector switches from LIN-45/Raf to RGL-1/RalGEF-RAL-1/Ral, we do not understand the mechanism of effector switching.

Isolated VPCs distal from the AC were originally shown to frequently assume 2° fate [20,35,36]. Yet since LIN-12 is essential for 2° fate induction by lateral signaling [24], it was unclear how these isolated VPCs were induced to become 2° cells. A resolution of this contradiction is that a combination of low dose LIN-3 and autocrine signaling by DSL Notch ligands could induce distal and isolated VPCs to assume 2° fates [72]. While it is unclear how this signaling mechanism intersects with sequential induction and morphogen gradient signaling mechanisms, a plausible model is that all three mechanisms collaborate to spatially induce, reinforce, and restrict 2° fate induction, thereby increasing patterning fidelity.

### 2.5. Wnt Signaling

Prior to induction, Wnt plays a critical role in establishing the competency of the P3.p-P6.p to respond to inductive signal, i.e., to become VPCs [73,74,75,76]. Wnt signaling also plays a central role in polarizing the 2° lineages of P5.p and P7.p to orient toward the AC and the 1° lineage [7,77,78,79]. A role for Wnt has also been found in VPC induction [74,75], but this role is difficult to untangle because the same mutations alter competency and polarity. To further complicate matters, Wnt signaling controls both competency and 3° cell fusion, and blockade of 3° cell fusion can potentiate inductive signals [80,81]. Consequently, the distinction between permissive and inductive roles of Wnt in VPC development are unclear [76].

## 3. Trafficking-Dependent Regulation of Receptor Localization and Function

In addition to the importance of intercellular spatial relationships in VPC fate patterning, intracellular spatial localization of signals within VPCs has been found to have critical importance. After LET-23 was identified to be an ortholog of the EGF receptor [31] and function cell autonomously in the presumptive 1° cell, P6.p [42,58,82], over-expressed GFP fusion proteins and antibody staining suggested that LET-23 is localized to the plasma membrane [83,84,85]. Subsequent analysis with lower copy number transgene *zhIs35[let-23::GFP]* suggests that LET-23 expression is dynamically regulated during VPC patterning [86]. These observations argue that subcellular localization of LET-23/EGFR and perhaps LIN-12/Notch provide key regulatory axes to control the VPC fate patterning signaling network.

### 3.1. LET-23 Basolateral Localization System

The *C. elegans* VPCs are polarized epithelial cells that are connected by adherens junctions [87]. Through these junctions, the six VPCs are tightly connected in the ventral midline in a single row. In addition, the cell junctions generate separated spatial domains of each VPC: the apical and basolateral plasma membranes of each VPC. Lipids and transmembrane proteins are potentially segregated by these adherens junctions, thus creating potentially distinct signaling domains.

Localization of LET-23 to the basolateral plasma membrane of the VPCs is necessary for 1° fate induction. A critical genetic tool for this discovery was the *let-23(sy1)* mutation, which introduces a premature stop that truncates the last six residues of the receptor. *let-23(sy1)* animals are vulvaless through lack of 1° induction, but are unaffected for other phenotypes regulated by LET-23, like development of the excretory duct cell or fertility [31,88]. The *sy1* mutation causes LET-23 to be mis-localized to the apical membrane of the VPCs, suggesting that the 1°-promoting signal occurs at the basolateral surface, closest to the AC [84]. Mutations in *lin-2*, *lin-7*, and *lin-10* similarly caused a Vul phenotype without impacting other LET-23 dependent developmental events. LIN-2, LIN-7, and LIN-10 encode orthologs of CASK, Veli, and Mint, respectively, and form a protein complex to localize LET-23 to the basolateral membrane of the VPCs. Of particular note is LIN-7, whose PDZ domain may recognize the PDZ recognition sequence in the C-terminus of LET-23 that is removed by the *let-23(sy1)* mutation to confer inappropriate apical localization of LET-23. Taken together, these results indicate that the 1°-promoting signal of LET-23 occurs at the basolateral surface, and requires the LIN-2/-7/-10 complex for proper localization.

A genetic screen for the identification of genes required for proper localization of LET-23::GFP identified ERM-1 (Ezrin/Radixin/Moesin). ERM-1 may function to keep LET-23::GFP sequestered in basolateral compartments, thus influencing trafficking, and ERM-1 is thought to function independently of LIN-2/-7/-10 [86]. Thus, multiple axes of spatial regulation likely impact LET-23 signaling.

### 3.2. Negative Regulators of LET-23 Function through Endocytosis, Trafficking, and Degradation

A series of negative regulators of the LET-23 1°-promoting signal may control endocytosis and intracellular trafficking of LET-23. Reduced function alleles of *unc-101* were discovered as suppressors of the *let-23(sy1)* vulvaless phenotype [89]. UNC-101 encodes a medium chain of the clathrin-associated complex AP-1. In a computational screen for genes with LAG-1 binding sites that are putative LIN-12 transcriptional targets, DPY-23 was found to antagonize the 1°-promoting signal [39]. DPY-23, which is a subunit of the clathrin Adaptor Protein Complex 2 (AP-2), has also been implicated in endocytosis of other signaling cascades, including Wnt [90].

*C. elegans* AGEF-1 is homologous to mammalian BIG1 and BIG2 ArfGEFs (guanine nucleotide exchange factors for the Arf family of small GTPases), which are involved in secretory trafficking between *trans*-Golgi, endosomes and plasma membrane through AP-1 recruitment [91,92,93,94,95]. Mutant *C. elegans* AGEF-1 suppressed the vulvaless phenotype of *let-23(sy1)* and *lin-2(e1309)*, suggesting that AGEF-1 functions as a negative regulator of LET-23 signaling; ARF-1.2 and ARF-3, potential GTPase substrates of AGEF-1, are also implicated as negative regulators of LET-23 [95]. The apical mis-localization of LET-23 in the *lin-2* mutant is partially restored by the *agef-1* mutant. This result suggests that AGEF-1 represses LET-23 basolateral localization in VPCs.

Mammalian Rab5 and Rab7, Rab family small GTPases, regulate early endosome and late endosome, respectively [96]. Rab5 promotes EGFR internalization, while Rab7 regulates EGFR trafficking from late endosomes to lysosomes [97,98,99,100,101]. The ortholog of mammalian Rab7, *C. elegans* RAB-7 was shown to be a negative regulator of LET-23: the *rab-7* mutant suppressed the vulvaless phenotype of *let-23(sy1)* and *lin-2(e1309)* [102]. In the *rab-7*; *lin-2* double mutant, LET-23::GFP is localized at both the apical membrane and the basolateral membrane in P6.p. Also, the LET-23::GFP is accumulated in endocytic vesicles, suggesting that RAB-7 regulates LET-23 trafficking. Loss of function of the dynein heavy chain, DHC-1, similarly suppressed the vulvaless phenotype of *let-23(sy1)* and *lin-2(e1309)*, suggesting that DHC-1 also represses basolateral trafficking of LET-23 [103].

Mutations in SLI-1 (Suppressor of Lineage defect) were identified as suppressors of the vulvaless phenotype of *let-23(sy1)* [104,105]. SLI-1 is the *C. elegans* ortholog of *Drosophila* D-Cbl and the mammalian proto-oncogene, c-Cbl [105], and its paralogous relatives Cbl-b and Cbl-c [106]. Mammalian c-Cbl functions as an E3 ubiquitin ligase that interacts with a broad set of signaling proteins harboring a phospho-tyrosine consensus sequence, most notably the EGFR [107,108]. A plausible target site by which SLI-1 inhibits LET-23 is through binding to putative phospho-tyrosine site 2 (out of 8 in the LET-23 cytoplasmic region), which has been shown to be a negative regulatory site [109]. Whether this negative regulation is via degradation or subcellular trafficking is unknown.

ARK-1 encodes the Ack-related cytoplasmic tyrosine kinase containing SH3 and CRIB (Cdc42/Rac interactive binding) domains [110]. The ARK-1 mutant suppresses vulvaless phenotype in *let-23(sy1), lin-2*, *lin-7*, and *lin-10* mutants and confers a synthetic Muv phenotype in double mutant combinations with mutations in *sli-1* or *unc-101*, suggesting that ARK-1 redundantly inhibits LET-23 [110]. The *ark-1* gene was identified as a potential transcriptional target of LIN-12 [39], suggesting that ARK-1 antagonizes LET-23 specifically in 2° cells, perhaps to prevent inappropriate 1°-promoting signal in 2° cells.

### 3.3. Regulation of LIN-12 Activity.

As noted above, LIN-12 protein is initially uniformly expressed in all VPCs and localized to the apical plasma membrane, then is internalized and degraded in the presumptive 1° cell after induction [59,61]. LIN-12 is expressed only in 2° cells and their daughter cells after the VPC fate specification.

Notch receptors are atypical, in that they comprise the entirety of their signal transduction cascade, from the plasma membrane to the nucleus. Specifically, upon ligand binding and activation, a series of proteolytic cleavage events releases the intracellular domain (ICD) of both *C. elegans* LIN-12 and *Drosophila* Notch receptors, which then translocates to the nucleus. There, the ICD functions as a transcriptional co-activator ([111]; reviewed in [66,112]).

LIN-12 was instrumental in defining components of Notch activation across species. Vertebrate and perhaps *C. elegans* Notch proteins are proteolytically cleaved at a “site 1” sequence in the extracellular domain [113,114]. The SUP-17/ADAM metalloprotease is required for functional LIN-12 signaling [115,116], and may act redundantly with the ADM-4 protease to cleave at an extracellular “site 2” [117]. Extracellular domain shedding leads to intracellular proteolytic cleavage at “site 3” by a proteolytic complex termed “γ-secretase,” in whose identification genetic analyses of LIN-12 was critical. Redundant SEL-12 and HOP-1 are presenilins, associated in humans with early onset Alzheimer’s [118], are critical for LIN-12 activation [119,120,121]. The details of γ-secretase regulation of LIN-12 and Notch receptors is complex, and involves developmental events other than VPC fate patterning.

SEL-2 has been shown to be a negative regulator of LIN-12 in 1° cell. In the SEL-2 mutant, LIN-12 was localized at the basolateral membrane in VPCs, indicating that SEL-2 regulates endocytic trafficking of LIN-12 [120]. *sel-10* encodes an FBW-like E3 ubiquitin ligase that negatively regulates LIN-12 [121]. However, SEL-10 also represses LIN-45, complicating interpretation and raising the question of whether SEL-10 generally represses vulval induction [12]. A wide array of additional positive and negative regulators of LIN-12 function have been described (reviewed in [66,112]).

## 4. Upstream and Downstream Transcriptional Regulators in VPC Fate Patterning

Changes in transcriptional regulation is central to many developmental processes. In VPC induction, controlled expression of the LIN-3 ligand in the AC patterns the VPCs, which have themselves undergone a prolonged developmental program that includes migrations and competency [2]. Downstream of inductive signaling lie transcriptional events that execute initial 1°- and 2°-specific fate programs. Here we briefly review known transcriptional programs upstream and downstream of the VPC signaling network.

### 4.1. Upstream: Repression of LIN-3 Expression by the SynMuv Genes

The synthetic multivulva (synMuv) phenotype was discovered by accident in screens for defective vulval formation: mutations in two synMuv genes is required to confer a Muv phenotype, while single mutations in either gene do not perturb vulval induction [22]. Initial examples discovered by accident were the *lin-8*; *lin-9* and *lin-15A/B* double mutants, each of which were shown to comprise mutations in two distinct genes. The synMuv classes A and B were subsequently populated by further screens for the synMuv phenotype in non-Muv single mutants [27,122,123,124]. Subsequent analyses found many more synMuv genes, and argued that even double mutants among the class B mutants confer the synMuv phenotype at high temperature [125]. Some synMuv genes may fall into a third class, Class C [123].

SynMuv genes are thought to antagonize LIN-3/EGF-LET-23/EGFR signaling. The synMuv mutant combination conferred a Muv phenotype that was suppressed by reduction of LET-23 function [23]. Early genetic mosaic experiments suggest that the synMuv *lin-15A/B* genes function in the hypodermal/epithelial cells surrounding the VPCs, leading to the model that the collection of synMuv genes defined a third pathway that inhibited vulval induction [126,127,128]. Consistent with these results, mosaic analysis and use of heterologous promoters indicated that the *lin-35* synMuv gene functions in hypodermis to repress vulval induction [126]. Critically, depletion of *lin-3* by RNAi demonstrated that the phenotype caused by mutation of synMuv genes requires LIN-3. Mutation of synMuv genes increases LIN-3 expression in hypodermal cells, and ectopic expression of LIN-3 from hypodermal cells was sufficient to confer a Muv phenotype [129]. Of critical importance was the identification of a dominant synMuv A group mutation, *lin-3(n4441)*, in the promoter of *lin-3.* smFISH experiments indicated that transcription of *lin-3* is tightly regulated spatially, but in synMuv mutants is derepressed, showing *lin-3* transcript expression in the surrounding hypodermal cells [130]. Consequently, a consensus model has emerged that the synMuv genes function collectively to repress the promoter of the *lin-3* gene, thus spatially restricting LIN-3 expression to the AC and robustly limiting the inductive signal to a precise point source.

Accordingly, many synMuv genes encode transcriptional and/or epigenetic regulators (reviewed in [131]). For example, some synMuv A group genes encode proteins that contain a zinc-finger-like THAP domain [132,133,134]. The synMuv B group genes have homology with mammalian proteins that are involved in chromatin remodeling, transcription repression, and histone modification [135,136,137,138,139,140,141]. A combination of direct transcriptional repression and gene epigenetic repression is thought to impose strict spatial restriction of the LIN-3 inductive signal. Less well understood is the role of four LIN-3 splice variants/isoforms and the potential role of the ROM-1/Rhomboid protease in propagation of the inductive signal [142,143,144].

### 4.2. Downstream: 1°- and 2°-Promoting Transcriptional Complexes

Screens for mutants conferring a Muv defect identified the genes *lin-1* and *lin-31*. By genetic epistasis both were found to function downstream in the 1° induction signaling cascade [23,145,146]. LIN-1 is an ETS/ELK-1-like transcription factor, which is frequently found as a downstream ERK target in mammalian cells [147]. Strong *lin-1* alleles confer an excess 1° phenotype that is insensitive to upstream pathway activity, leading to the model that MPK-1/ERK represses LIN-1 activity, which in turn represses 1° fate. This model was validated by gain-of-function mutations in *lin-1* that confer a vulvaless phenotype, and which identify C-terminal repressive MPK-1/ERK phosphorylation sites [148,149]. The transcriptional targets of 1°-promoting signaling are *egl-17* and DSL ligands of LIN-12/Notch encoded by *lag-2*, *apx-1*, and *dsl-1* [29,38].

LIN-31 is a winged helix transcription factor orthologous to mammalian HNF-1 and *Drosophila* Forkhead ([146]; in modern nomenclature, FoxB). Similar to LIN-1, LIN-31 is also phosphorylated by MPK-1/ERK, and a putative LIN-31-LIN-1 heterodimer is disrupted by this phosphorylation. Over-expression of non-phosphorylatable LIN-31 repressed vulval fates, consistent with this model [150]. However, subsequent CRISPR knock-ins of phosphodefective and phosphomimetic mutations in the same putative MPK-1 sites failed to alter VPC patterning, so regulation of LIN-31 may be more complex [151]. Yet in contrast to LIN-1, disruption of LIN-31 function confers both Muv and Vul phenotypes: the vulval lineages of *lin-31* mutants could be described as randomized, with any VPC assuming any fate [146,150,152]. Consequently, LIN-31 is perceived as a critical determinant of all three potential VPC fates, but its regulation and interactions with other transcriptional machinery is still not understood.

SUR-2/Med23 and LIN-25/Med24 are important for 1° fate induction and were identified, respectively, based on suppression of activated LET-60/Ras and a Vul phenotype. SUR-2 and its partner LIN-25 are subunits of the multi-subunit transcriptional Mediator complex, and function downstream of or parallel to MPK-1 in VPCs [28,63,153,154]. Through use of diverse cofactors to generate a variety of distinct complex types, the Mediator complex functions to bridge tissue-specific transcription factors and RNA polymerase II, as well as potentially integrating inputs of various transcriptional enhancers and repressors [155]. Mammalian Elk1, an ortholog of LIN-1, interacts with the MED23/Sur2 in an ERK-dependent manner [156], validating the model of MPK-1 repression of LIN-1 and the role of SUR-2/LIN-25 and the Mediator complex in VPC induction. SUR-2 and the Hox protein LIN-39, which is required for VPC competence [157,158,159], likely collaborate to promote transcription of the lateral signaling genes *lag-2*, which encodes a DSL ligand for LIN-12/Notch [160,161]. Genetic analyses suggest that various subtypes of the Mediator complex, particularly the CKM module, function to set activity thresholds and discriminate between MPK-1/ERK 1°- and LIN-12/Notch 2°-promoting signaling activity, thus providing a key integration point between the VPC signaling network and precise transcriptional execution of VPC fates [157,162]. In parallel to these transcriptional mechanisms are EOR-1 and EOR-2 (EGL-1 suppressor, Di-O uptake defective, *raf* enhancer), which also function together downstream of MPK-1 to positively regulate vulva induction [163].

A key advance in *C. elegans* Notch biology was the discovery that the two nematode Notch receptors, GLP-1 and LIN-12, share functional redundancy in certain processes, and are even functionally interchangeable [164]. The double mutant conferred a distinctive first stage (L1) larval arrest dubbed the LAG phenotype (LIN-12 and GLP-1; [165]. Screens for this phenotype identified two additional genes in the Notch system, LAG-1 and SEL-8/LAG-3 (LAG-2 encodes a shared LIN-12 and GLP-1 DSL ligand; [160,161]). LAG-1 encodes the nematode ortholog of *Drosophila* Suppressor of Hairy (Su(H)) and mammalian CBF1, established DNA-binding proteins. Like Su(H)/CBF1, LAG-1 binds a conserved consensus target sequence, RTGGGAA [166]. LAG-1 binds the LIN-12 ICD, and together they can activate transcription [167]. SEL-8/LAG-3 is a Glutamine-rich protein, similar to *Drosophila* mastermind that forms a complex with LAG-1 [168,169]. Together, these proteins and the ICD likely form a ternary complex that regulates transcription of tissue-specific client genes.

Using a more refined consensus binding sequence from other systems (YRTGTGAA; “Lag binding sequence (LBS)”) potential target genes of Notch signaling were identified computationally. Candidates were validated by RNAi depletion and promoter::GFP transcriptional fusions Thus, the Notch target genes such as *dpy-23*, *lst-1*, -*2*, -*3*, -*4*, and *mir-61* were identified. Collectively, these genes appear to function to antagonize 1°-promoting signals, contributing to the Mutual Antagonism Model. Target genes that promote 2° fate have not yet been identified, suggesting that they share redundant functions [39,40].

## 5. Transcriptional Reprogramming of the VPC Signaling Network

Prior to induction of naïve VPCs, expression of promoter::GFP fusions is typically uniform. Soon after induction, however, GFP expression levels from many promoters, particularly of modulatory genes, is dynamically regulated. This reprogramming contributes to key mechanisms of VPC fate patterning, such as mutual antagonism (see above), and thus represents plasticity of the signaling network that accompanies initial specification of fates. Furthermore, we posit that reprogramming of the expression of modulatory signals helps reinforce initial patterning, so VPCs can commit to their fate decisions while mitigating conflicting signals, or “noise”, which could introduce developmental error.

Mostly, transcriptionally reprogrammed genes have been identified as negative regulators of the 1°-promoting signal, or computationally discovered as transcriptional targets of the LIN-12/Notch lateral signal. Whether the latter are 2°-promoting or anti-1° is difficult to determine by existing genetic assays. The restriction of gene expression to specific VPC lineages may reinforce the final commitment and fidelity during VPC fate patterning. Here we describe the examples of transcriptional reprogramming in VPC fate patterning.

**Figure 4:**
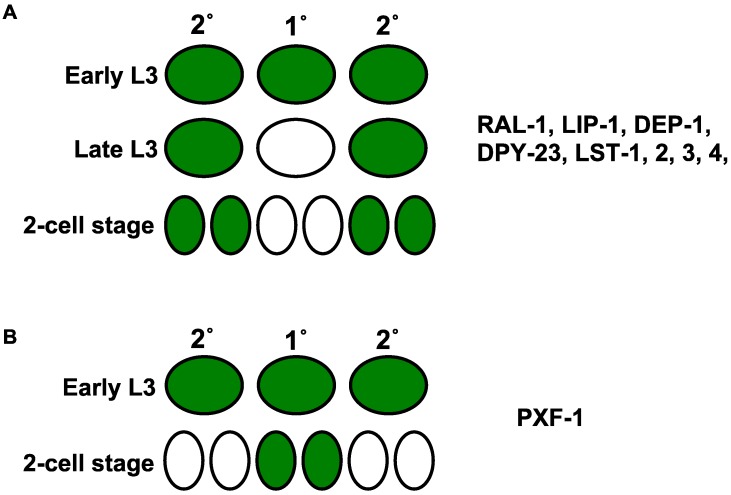
Reprogramming expression from transcriptional GFP reporters of either 2°-promoting/1°-antagonizing or 1°-promoting genes. Transcriptional reprogramming is shown by promoter GFP fusion in VPCs. (**A**) Transgenic promoters of 2°-promoting *ral-1* and 1°-antagonizing Notch transcriptional targets *lip-1*, *dep-1*, *dpy-23*, *lst-1*, *-2*, *-3*, and *-4*, express GFP in both presumptive 1° and 2° cells at early L3, before induction. After induction, GFP expression is reduced in 1° but persists in 2° lineages. (**B**) The transgenic promoter of 1°-promoting *pxf-1* expresses GFP in presumptive 1° and 2° cells at early L3, before induction. After induction, GFP persists in the the 1° lineage but is reduced in the 2° lineage. Thus, expression of modulatory genes in the VPC fate patterning network are reprogrammed during the inductive process.

Reporters for LIN-12/Notch target genes are expressed uniformly in VPCs at the early L3 stage, before VPC fate patterning has happened. Strikingly, later in the L3, typically before the first cell division, expression from reporters is excluded from the 1° but not the 2° cell/lineage. Reporters are expressed strongly in 2° cells after VPC fate patterning (Figure 4A). For example, the 2°-promoting *ral-1* gene, LIN-12/Notch target genes *dpy-23*, *lst-1*, *2*, *3*, and *4*, and 1°-antagonizing genes *lip-1* and *dep-1* all showed this general transcriptional expression pattern in VPCs [37,39,64,69]. We speculate that such transcriptional reprogramming of these genes reinforces 1°-antagonizing and/or 2°-promoting function, thereby better demarcating cell fate signaling and increasing developmental fidelity in the system.

An exception to this observation is highlighted by the ligands for the lateral 2°-promoting signal mediated by LIN-12/Notch: transcriptional reporters for *apx-1*, *dsl-1*, and *lag-2* are expressed in the presumptive 1° cell in response to inductive signal and repressed in non-1° cells [29,57]. Their reporter expression reflects a pattern otherwise expected for 1°-promoting genes, which is consistent with their cell non-autonomous role as the ligands for the LIN-12/Notch lateral signal. This observation is an important validation of the Sequential Induction Model, and may be the exception that proves the rule for transcriptional reprogramming.

Transcriptional reporters for 1°-promoting genes also reveal initially uniform expression that dynamically changes after induction. However, in this case the induction reflects reprogramming consistent with initial specification to promote 1° fate (Figure 4B). A different modulatory signaling axis, that of PXF-1/RapGEF signaling to the closely related LET-60/Ras sibling, RAP-1/Rap1, promotes 1° fate in parallel to LET-60 [170]. CRISPR-tagged endogenous RAP-1 is expressed ubiquitously, and GFP expressed from a transgenic promoter fusion of *pxf-1* showed uniform expression in all VPCs at early L3 stage before VPC fate patterning. However, after induction, *pxf-1* reporter GFP expression was excluded from the 2° cells and increased in the 1° cells at the Pn.px stage. This result is consistent with transcriptional reprogramming of PXF-1 expression restricting activation of 1°-promoting RAP-1 to the 1° cell while abrogating the activation of RAP-1 in 2° cells. Consequently, we hypothesize that PXF-1-RAP-1 functions as a spatially refined positive feedback loop to promote 1° fate.

Transcriptional reporters for the *egl-17* gene reveal an expression pattern that reflects the putative morphogen gradient: reporter expression is absent prior to induction, then after induction is high in presumptive 1° cells and faint and transient in presumptive 2° cells [38,39]. Mutations in *lip-1* and *dep-1*, negative regulators of the 1°-promoting cascade, cause the *egl-17* reporter to persist in 2° cells [37,39,64]. Yet this reporter is likely not a reporter for 1° fate, but rather a transcriptional output downstream of MPK-1/ERK [38]. EGL-17 is an ortholog of mammalian FGF, and its secretion by the presumptive 1° cell helps the migrating SM cells home in on the A-P midpoint of the animal [171].

The regulation of genes at the transcriptional level does not necessarily reflect expression of protein at the translational level. For example, the promoter::GFP transcriptional fusion of *ral-1* showed dynamic changes in levels during VPC induction [69]. In contrast, the endogenously tagged RAL-1 by CRISPR appears to be expressed uniformly throughout VPC development, and localized to the plasma membrane in all VPCs [71]. How do we reconcile these differences? One possibility is that transgenic promoter fusions mis-express GFP in a pattern that does not reflect endogenous protein expression. Transcriptional changes may not dramatically impact stable endogenous protein with low turnover. However, transcriptional changes coupled with other layers of post-transcriptional and post-translational changes may still collectively impact signaling outputs, and thus restrict signaling activity to certain cell types and exclude signals from others. Thus, the observed transcriptional reprogramming may be functionally significant in concert with other regulatory modalities.

## 6. Environmental and Genetic Regulators of Variability

Changes in the environment could represent one of the main perturbations to the fidelity of a developmental system. Errors in VPC patterning can result in decreasing progeny. Thus, the result of VPC fate patterning is related to reproductive success and evolutionary fitness. The *C. elegans* VPC fate patterning is a precise and robust process. The VPC fate patterning has 99.8% rate of accuracy with variable environmental conditions [16]. The fidelity of VPC fate patterning is controlled by signaling network, mainly 1°-promoting Ras and 2°-promoting Notch signals. Therefore, perturbation of these signals can provoke variation of VPC fate patterning with increased error rate [16]. This system has also been used to assess the impact of heterogeneity in polymorphic wild *C. elegans* isolates [19,172]. While mutation perturbation of the balance of 1° vs. 2° signaling axes increases sensitivity to environmental perturbations, basal signaling error is not increased [173,174,175,176].

## 7. Conclusions

A view is emerging of a sophisticated signaling network that controls the fate patterning of the *C. elegans* VPCs. In response to LIN-3/EGF, VPCs are precisely patterned by two main signaling cascades. The necessary and sufficient EGFR- and Notch-mediated signals establish the core pattern of initial fate specifications. A signaling gradient, orchestrated modulatory signaling cascades, and transcriptional reprogramming of mutual antagonism induction programs act together to further sculpt these fate decisions both spatially and temporally. Collectively, these mechanisms collaborate to generate a highly precise and robust pattern prior to terminal differentiation. Most of the signaling molecules described in the VPC patterning network are conserved in mammals as proto-oncogenes or tumor suppressor genes. Thus, study of the VPC patterning system provides insights into our understanding of signaling networks in both development and pathology.
